# Boosting Long-Term Memory via Wakeful Rest: Intentional Rehearsal Is Not Necessary, Consolidation Is Sufficient

**DOI:** 10.1371/journal.pone.0109542

**Published:** 2014-10-15

**Authors:** Michaela Dewar, Jessica Alber, Nelson Cowan, Sergio Della Sala

**Affiliations:** 1 Department of Psychology, School of Life Sciences, Heriot-Watt University, Edinburgh, United Kingdom; 2 Human Cognitive Neuroscience, Department of Psychology, University of Edinburgh, United Kingdom; 3 Centre for Cognitive Ageing and Cognitive Epidemiology, Psychology, University of Edinburgh, Edinburgh, United Kingdom; 4 Department of Psychology, University of Missouri, Columbia, Missouri, United States of America; The University of Chicago, United States of America

## Abstract

People perform better on tests of delayed free recall if learning is followed immediately by a short wakeful rest than by a short period of sensory stimulation. Animal and human work suggests that wakeful resting provides optimal conditions for the consolidation of recently acquired memories. However, an alternative account cannot be ruled out, namely that wakeful resting provides optimal conditions for intentional rehearsal of recently acquired memories, thus driving superior memory. Here we utilised non-recallable words to examine whether wakeful rest boosts long-term memory, even when new memories could not be rehearsed intentionally during the wakeful rest delay. The probing of non-recallable words requires a recognition paradigm. Therefore, we first established, via Experiment 1, that the rest-induced boost in memory observed via free recall can be replicated in a recognition paradigm, using concrete nouns. In Experiment 2, participants heard 30 non-recallable non-words, presented as ‘foreign names in a bridge club abroad’ and then either rested wakefully or played a visual spot-the-difference game for 10 minutes. Retention was probed via recognition at two time points, 15 minutes and 7 days after presentation. As in Experiment 1, wakeful rest boosted recognition significantly, and this boost was maintained for at least 7 days. Our results indicate that the enhancement of memory via wakeful rest is *not* dependent upon intentional rehearsal of learned material during the rest period. We thus conclude that consolidation is *sufficient* for this rest-induced memory boost to emerge. We propose that wakeful resting allows for superior memory consolidation, resulting in stronger and/or more veridical representations of experienced events which can be detected via tests of free recall and recognition.

## Introduction

A period of wakeful rest immediately after new learning boosts free recall of verbal material. Several studies show that both young and elderly people recall more newly learned verbal material after a 10-60-minute interval if this interval is filled with wakeful rest than with sensory stimulation [1]–[4]. A recent study showed that this memory enhancement via a brief wakeful rest is maintained for at least 7 days in healthy elderly people [2]. Here we investigate the cognitive basis of this memory boost.

Recent insights from human and animal neuroscience suggest that wakeful rest improves memory by enhancing memory consolidation [2], [5]–[11]. Memory consolidation is defined as the automatic process by which memories strengthen over time [12]. Evidence for this consolidation process comes from animal work showing that, over time, new memories become less susceptible to the interfering effects of pharmacological manipulations [12]–[14]. In keeping with these animal findings, our work in humans shows that new memories are retained better if post-learning cognitive tasks are delayed than if they take place immediately after learning, i.e. a temporal gradient of behavioural interference is observed [2], [3].

Research in rodents suggests that consolidation is associated with the spontaneous reactivation of recent encoding-related neural activity, and that this reactivation occurs predominantly during states of relative immobility, such as sleep and wakeful rest [8], [9], [15], [16]. Recent neuroimaging work in humans strengthens this consolidation hypothesis, revealing (i) reactivation of recent encoding-related neural activity during wakeful rest, and (ii) a direct link between the degree of such reactivation and performance on subsequent memory [10], [11]. This work in animals and humans suggest that wakeful rest provides optimal conditions for consolidation of recently acquired memories, perhaps due to minimal encoding of novel interfering information [2], [6], [7].

However, an alternative account cannot be ruled out, namely that wakeful resting provides optimal conditions for *intentional rehearsal* of recently acquired memories, thus driving superior memory during wakeful rest delays, at least in humans. Intentional retrieval of learned material improves long-term retention of newly learned material [17], [18], and this ‘retrieval practice’ effect is observed when participants rehearse material overtly or covertly [19]. Elaborative rehearsal, the intentional integration of newly acquired memory traces within an existing framework in long-term storage, also enhances long-term free recall and recognition memory [20]–[22].

Could intentional rehearsal be at the heart of the memory boost observed via post-learning wakeful rest? Several findings speak against this possibility: firstly, in the research on wakeful rest and memory, participants are not informed about the delayed recall test, thus reducing the motivation to intentionally rehearse the memoranda in order to augment test performance [1]–[3]. Secondly, during structured post-experimental debriefing, the majority of participants report that they did not think about test material during the wakeful rest period [2], [3], [11]. However, this evidence is derived indirectly rather than via controlled experiments, and therefore the intentional rehearsal hypothesis cannot be dismissed.

Here, we report two experiments, in which we explored whether wakeful resting boosts verbal long-term memory, even when new memories *cannot* be rehearsed *intentionally* during the rest interval. To this end, we aimed to use non-recallable non-words as our memoranda. The use of non-recallable words requires the application of a recognition paradigm. Therefore, our first experiment examined whether the rest-related boost in memory that is observed in free recall [1]–[3] is also observed in recognition when using common nouns. Our second experiment used a recognition paradigm to examine whether wakeful rest improves long-term recognition of non-words (e.g., *phiefnierds*) that could not be recalled freely.

Results indicating that even non-recallable stimuli can benefit from a post-learning interval of wakeful rest would refute the hypothesis that intentional rehearsal is necessary to achieve a long-lived memory benefit via wakeful rest.

## Experiment 1

### Methods

#### Ethics statement

Both experiments were approved by the University of Edinburgh's Psychology Research Ethics Committee (Ref: 187-1112/1; 190-1213/1). All participants provided their informed consent in writing prior to taking part in our research.

#### Participants

We tested healthy elderly people since we sought to follow up our previous findings involving this population [2]. Healthy elderly people constitute a growing portion of the population and often report age-related difficulties with memory [2]. Seventy healthy volunteers (21m/49f) were randomly divided into two groups, based on post-learning stimulation condition: *high sensory stimulation* (N = 36, mean age = 73 years, age range = 62–83 years; mean NART-predicted IQ = 119.29, range = 105.96–127.17, [23]; 8m/28f) and *minimal sensory stimulation* (N = 34, mean age = 71 years, age range = 61–87 years; mean NART-predicted IQ = 118.04, range = 106.74–125.81, [23]; 13m/21f).

Participants had no premorbid neurological or psychiatric history, and demonstrated normal scores on a thorough neuropsychological test battery, including the Addenbrooke's Cognitive Examination-Revised (ACE-R) [24]. The ACE-R is a widely-used screening test for cognitive impairment. We applied the conservative ACE-R cut-off of 88 to ensure that all of our participants were cognitively intact. The groups did not differ significantly in age (*p* = .147), NART-predicted IQ (*p* = .245) or ACE-R scores (*p* = .204).

#### Design

Experiment 1 included two testing sessions, Session 1 and Session 2, which were separated by 7 days. We used a mixed design to examine retention of a list of words. There were two key manipulations: stimulation condition (high sensory stimulation vs. minimal sensory stimulation; between subjects) and retention interval (15 minutes vs. 7 days; within subjects).

#### Materials

A total of 30 unrelated, common nouns (e.g. platform, daylight, specialist) were grouped into two lists of 15 words each for memory testing. The words were selected from the MRC Psycholinguistic database and were matched for number of letters, syllables, familiarity, concreteness, imaginability and frequency (frequency was taken from the British National Corpus). One word list was used as the target list, the other as the foil list. The target and foil lists had a similar range in number of letters, syllables, familiarity, concreteness, and imaginability, but they did not overlap highly in terms of semantics or phonology, i.e. the foils were not highly similar to the targets.

30 picture pairs were employed as filler materials. The picture pairs were photographs of complex real-world scenes (e.g. landscapes, animals and people). They were manipulated so that the pictures in each pair differed in 2 subtle ways [2].

#### Procedure

**Figure 1:**
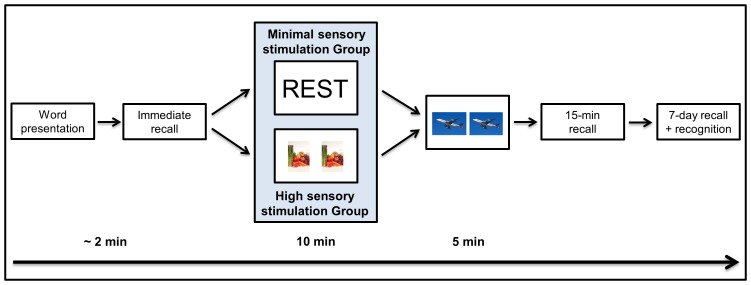
Participants were assigned randomly to the high sensory stimulation (N = 36) or minimal sensory stimulation (N = 34) group. In Session 1, all participants were presented with a list of 15 common nouns, which they were told to remember for subsequent immediate free recall. Following this, participants in the high sensory stimulation group completed a spot-the-difference task for 10 minutes, whereas participants in the minimal sensory stimulation group rested wakefully for 10 minutes. All participants then completed a 5-minute distractor task (spot-the-difference), which was succeeded by a surprise delayed recall test of the word list. In Session 2, which took place 7 days after Session 1, participants completed another surprise delayed free recall of the word list, as well a 30-item yes/no recognition test, including a remember/know paradigm.

Figure 1 illustrates the procedure of Experiment 1. In *Session 1*, participants were presented with the list of 15 target words. The words were presented aurally to participants at a rate of 1 word per second, with a 2-second interval between words. Participants were asked to try to remember as many words as possible, in any order, for an immediate recall test. This was followed by a 10-minute delay filled with either (i) high sensory stimulation or (ii) minimal sensory stimulation. Participants were not informed about subsequent delayed recall at this time.

Participants in the *high sensory stimulation group* completed 10 minutes of a spot-the-difference task, during which they were presented sequentially with 30 picture pairs on a laptop screen [2]. Their task was to identify and point to two differences between each picture pair within a 20-second time limit. Participants were instructed not to talk during the task, and care was taken to ensure that the spot-the-difference task was entirely visual: full instructions as well as a 1-minute practice trial were administered prior to Session 1 in order to minimalize verbalization during the delay. The spot-the-difference task was employed for two key reasons: firstly, it introduced new meaningful material and was cognitively demanding, thereby hampering word list *consolidation* [1]–[4], [6]. Secondly, it was non-verbal and highly unlike the word lists, thereby minimising potential interference at *retrieval* between word list memories and filler task memories [1], [2]. That is, the visual spot-the-difference task allowed us to examine the effect of sensory stimulation condition on word list consolidation specifically, without the potential confound of retrieval interference.

Participants in the *minimal sensory stimulation group* were instructed to rest quietly in a darkened testing room while the experimenter went to ‘organize the next part of the study’ [2], [3]. To ensure minimal sensory stimulation, all equipment was turned off, and participants had no access to mobile phones, newspapers, etc.

In order to make sure that both groups engaged in identical activity prior to delayed recall, all participants completed a 5-minute spot-the-difference distractor task immediately after the 10-minute post-learning delay period [2]. Subsequent to this, participants were asked to recall orally as many of the 15 words as they could, in any order (15-minute delayed recall). To conclude Session 1, participants completed a structured post-experimental survey asking such questions (as applicable) as: ‘did you expect to be asked to remember the words again?’ and ‘did you think about the words during the wakeful rest delay?’ Participants were not informed of the nature of Session 2 at this juncture.

At the beginning of *Session 2*, which occurred 7 days after Session 1, participants received a *free recall test*: they were asked again to recall orally as many of the 15 words as they could, in any order (7-day delayed recall). Upon completion of the 7-day free recall test, participants performed an untimed *recognition test*: this was a 30-item yes/no test, comprising the 15 target words and 15 foils. Target and foil words were presented orally in the same random order for each participant. Participants were asked whether the word was old (i.e. had been presented in Session 1) or new (i.e. had not been presented in Session 1). If they made an ‘old’ response, participants were asked to make a remember/know judgment [25], [26]. Participants were instructed to respond ‘remember’ if their recognition of a word was accompanied by recollection of its occurrence a week earlier, and ‘know’ if a word ‘rings a bell’ but was not accompanied by any recollection of its occurrence a week earlier. They were given a number of examples prior to the remember/know test to ensure that they understood and were comfortable with the distinction between these two forms of memory. We applied the remember/know task to allow for a more fine-grained probing of the effect of wakeful rest on memory, in case this was not picked up adequately by a simple yes/no recognition test. The remember/know task is frequently used to differentiate between recollection (remember) and familiarity (know) [27]–[29], although there is a lively debate as to whether this apparent distinction between recollection and familiarity reflects separate memory processes (dual-trace theory) [27], [29]–[32] or simply reflects variations in a continuous memory strength (single-process theory) [33], [34].

At the end of *Session 2*, participants completed another post-experimental survey, asking whether they had thought about the words in the 7 days since Session 1, and whether they expected to be asked to recall the words again.

#### Scoring

For the *free recall test*, we computed the total number of words recalled correctly at (i) immediate recall, (ii) 15-minute delayed recall, and (iii) 7-day delayed recall. In order to discern how many words recalled at immediate recall were retained at 15-minute and at 7-day delayed recall, a percentage retention score was computed for each participant at 15-minute delayed recall and 7-day delayed recall [2], [3], [5], [35]–[37]. Percentage retention scores were calculated by dividing the number of words recalled at 15-minute and 7-day delayed recall by the number of words recalled at immediate recall, and multiplying this quotient by 100. All percentage retention scores were capped at 100%. Percentage retention scores control for individual differences and any between-group variation at immediate recall.

For the *recognition test*, hit rates were calculated by dividing the number of targets correctly identified by the total number of targets (/15). False alarm rates were calculated by dividing the number of foils incorrectly identified as targets by the total number of foils (/15). In order to measure recognition accuracy, d-prime (d′) was calculated via the following equation: *d′ = z(hit rate) – z(false alarm rate)*. None of the participants had a hit rate of 1, thus no corrections had to be made to hit rate scores during the computation of d′. 1 participant had a false alarm rate of 0, thus requiring correction for the computation of d′. In line with standard correction procedures, we corrected this score by adding half a false alarm to this score, i.e. (1/30), resulting in a corrected false alarm rate of 0.033. As a further measure of recognition accuracy, we calculated the correct response rate. This was computed by adding the number of targets correctly identified as targets (hits) and the number of foils correctly identified as foils (correct rejections) and dividing this number by the total number of targets and foils (/30).

#### Statistical analyses

The alpha level was set to.05 for all analyses, which were conducted in IBM SPSS Statistics 19. We compared the two sensory stimulation groups' immediate free recall via a one-way ANOVA. We analysed the free recall proportion retention data by carrying out a mixed model, repeated measures ANOVA with time as a within subjects factor (15 minutes vs. 7 days) and stimulation condition as a between subjects factor (high sensory stimulation vs. minimal sensory stimulation). Based on our previous findings [2], we ran two planned comparisons, using one-way ANOVAs, to examine whether minimal sensory stimulation improved proportion retention (i) after 15 minutes and (ii) after 7 days. We compared the sensory stimulation groups' 7-day recognition data via one-way ANOVAs. Furthermore, we ran repeated measures ANOVAs to compare within each group the proportion of hits ‘remembered’ and the proportion of hits ‘known’. Lastly, we used Pearson's correlations to examine associations between 7-day free recall percentage retention and 7-day recognition performance.

### Results

#### Immediate free recall

Immediate recall scores did not differ between the minimal sensory stimulation group (mean = 6.09, SEM  = .218) and the high sensory stimulation group (mean  = 6.06, SEM  = .219), (*F*(1, 68) = 0, *p* = .992). This indicates that baseline performance was matched for the stimulation condition groups.

#### Delayed free recall - Retention of word lists after (a) 15 minutes and (b) 7 days

**Figure 2:**
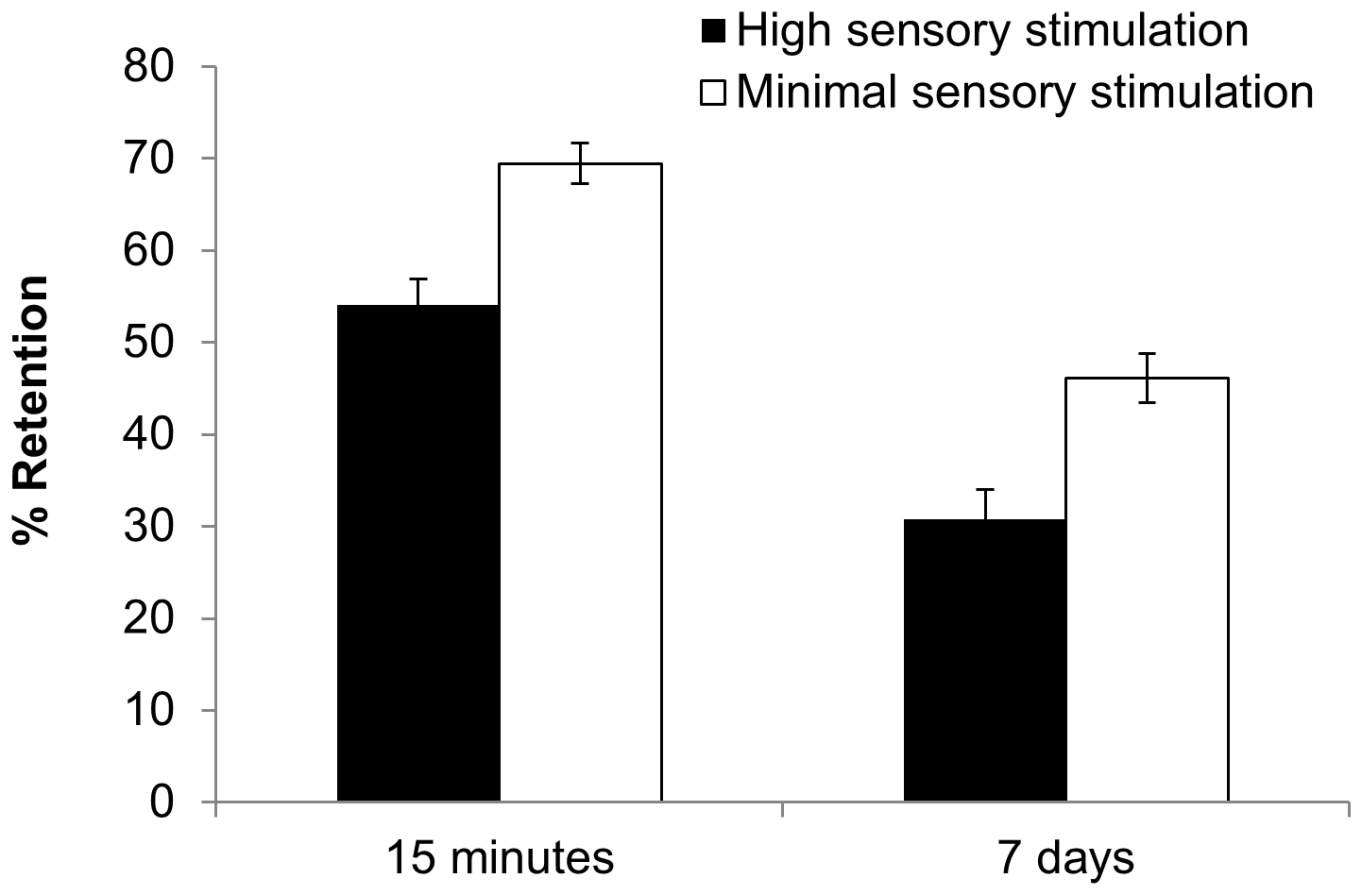
Mean percentage retention scores at 15-minute and 7-day delayed free recall ((Delayed/Immediate) x 100) in the high sensory stimulation and minimal sensory stimulation groups in Experiment 1. 15-minute retention was significantly higher in the minimal sensory stimulation group than in the high sensory stimulation group, and this benefit was maintained over 7 days. Error bars  =  standard error of the mean.

Figure 2 shows that 15-minute wordlist retention was significantly higher in the minimal sensory stimulation group than in the high sensory stimulation group, (*F*(1, 68) = 17.87, *p*<.001). Retention dropped over 7 days in both stimulation groups (*F*(1, 68) = 138.488, *p* <.001, η_p_^2^ = .671). However, the superior retention in the minimal sensory stimulation group relative to the high sensory stimulation group was maintained at 7-day delayed recall, (*F*(1, 68) = 12.957, *p*<.01), with no further additional benefit after 7 days, i.e. no significant time x group interaction (*F*(1, 68) = 0, *p* = .991, η_p_^2^ = 0).

Table 1 shows all data for the yes/no *word recognition* test (15 targets and 15 foils) taking place *after 7 days*.

**Table 1:** Mean recognition performance (+ SEM) of the minimal sensory stimulation groups (wakeful rest delay) and the high sensory stimulation groups (spot-the-difference delay) in Experiment 1 and 2.

Experiment 2							Experiment 1		
(non-words)							(common nouns)		
	15 minutes			7 days			7 days		
	Minimal	High	*p-*value	Minimal	High	*p*-value	Minimal	High	*p-value*
**Yes/No Test**									
**d'**	**1.209 (.091)**	**.696 (.103)**	**<.001**	**1.000 (.086)**	**.449 (.100)**	**<.001**	**1.032 (.084)**	**.757 (.078)**	**<.05**
**Correct response rate**	**.712 (.014)**	**.626 (.018)**	**<.001**	**.670 (.016)**	**.575 (.016)**	**<.001**	**.685 (.013)**	**.636 (.015)**	**<.05**
**Hit rate**	.677 (.026)	.625 (.033)	.227	.553 (.036)	.469 (.034)	.094	.661 (.019)	.633 (.022)	.352
**False alarm rate**	**.253 (.021)**	**.373 (.028)**	**<.005**	**.212 (.020)**	**.320 (.027)**	**<.005**	**.290 (.023)**	**.362 (.024)**	**<.05**
**Remember/Know**									
**Proportion Remember - Hits**	.348 (.039)	.290 (.032)	.255	.304 (.039)	.273 (.034)	.55	**.497 (.030)**	**.381 (.037)**	**<.05**
**Proportion Know - Hits**	.652 (.039)	.710 (.032)	.255	.696 (.039)	.727 (.034)	.55	**.503 (.030)**	**.619 (.037)**	**<.05**
**Proportion Remember - False alarms**	.079 (.026)	.071 (.026)	.832	.130 (.047)	.070 (.042)	.392	.049 (.031)	.041 (.022)	.833
**Proportion Know - False alarms**	.921 (.026)	.929 (.026)	.832	.870 (.047)	.924 (.042)	.392	.951 (.031)	.959 (.022)	.833

#### d′

d′ was significantly higher in the minimal sensory stimulation group than in the high sensory stimulation group, (*F*(1, 68) = 5.694, *p*<.05).

#### Correct response rate

Correct response rate (hits + correct rejections/30) was significantly higher in the minimal sensory stimulation group than in the high sensory stimulation group (*F*(1, 68) = 6.206, *p*<.05).

#### Hit rate and false alarm rate

Hit rate did not differ significantly between the high sensory stimulation group and the minimal sensory stimulation group (*F*(1, 68) = .878, *p* = .352). However, false alarm rate was significantly higher in the high sensory stimulation group than in the minimal sensory stimulation group (*F*(1, 68) = 4.477, *p*<.05).

#### Remember/Know

**Figure 3:**
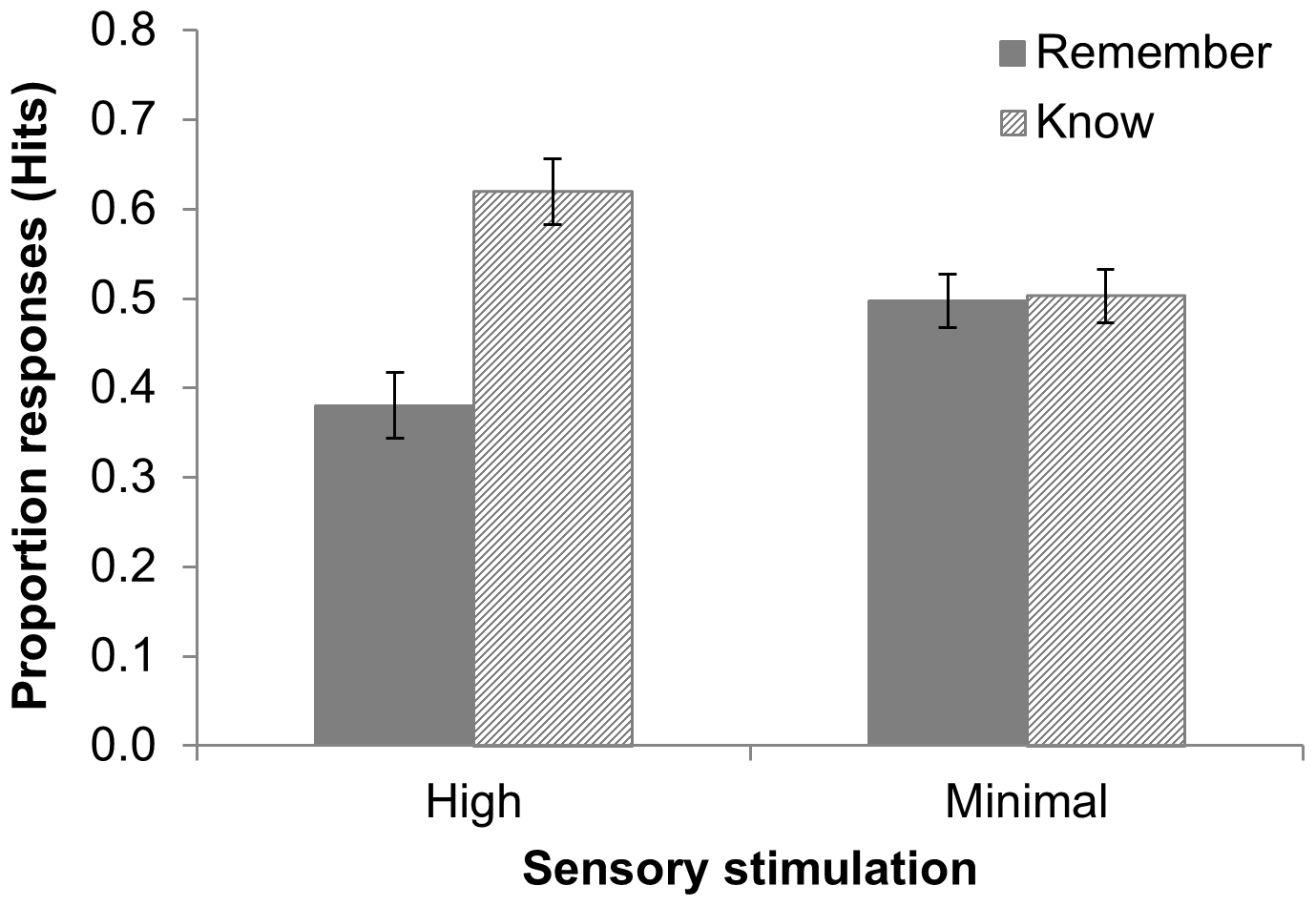
Proportion of ‘remember’ and ‘know’ responses for *hits* in the high sensory and minimal sensory stimulation groups after 7 days in Experiment 1. The proportion of ‘remember’ responses was higher in the minimal sensory stimulation than in the high sensory stimulation group. Error bars  =  standard error of the mean.

Figure 3 and Table 1 show that for hits, the proportion of ‘remember’ responses was significantly higher in the minimal sensory stimulation group than in the high sensory stimulation group (*F*(1, 68) = 5.857, *p*<.05). Correspondingly, the proportion of ‘know’ responses was significantly lower in the minimal sensory stimulation than in the high sensory stimulation group. Indeed, while in the minimal sensory stimulation group there was no significant difference in the proportion of ‘remember’ and ‘know’ responses (*F*(1, 33) = .009, *p* = .927, η_p_^2^ = 0), in the high sensory stimulation group, the proportion of ‘know’ responses was significantly higher than the proportion of ‘remember’ responses (*F*(1, 35) = 10.326, *p*<.01, η_p_^2^ = .228).

#### Associations between 7-day free recall and 7-day recognition performance (collapsed over both groups)

Percentage retention at 7-day free recall correlated significantly and *positively* with d′ (*r* = .322, *p* <.001), correct response rate (*r* = .350, *p*<.01), and with proportion of hits ‘remembered’ (*r* = .424, *p*<.001). Moreover, percentage retention at 7-day free recall correlated significantly and *negatively* with false alarm rate (*r* = −.326, *p*<.01), and with proportion of hits known (*r* = −.424, *p*<.001). However, percentage retention at 7-day free recall did not correlate significantly with hit rate (*r* = .107, *p* = .377).

#### Post-experimental questionnaire

The questionnaire data revealed the following:

*Expected recall* - 1 participant in the high sensory stimulation group and 3 participants in the minimal sensory stimulation group reported that they had expected delayed recall in Session 1. Six participants in the high sensory stimulation group and 10 participants in the minimal sensory stimulation group reported that they had expected delayed recall in Session 2; *Delay activity and rehearsal* - four participants in the minimal sensory stimulation group reported thinking about the words during some of the wakeful rest period. The other participants reported mind-wandering. Ten of the participants in the high sensory stimulation group and 11 participants in the minimal sensory stimulation group reported thinking about the words during the 7-day interval (1–6 times).

We repeated the above analyses, including only those participants who did not report thinking about the words (during the wakeful rest period and/or between sessions) or expecting delayed recall (high sensory stimulation group N = 24, minimal sensory stimulation group N = 20). The results did not change, bar the group difference in 7-day hit rate (minimal sensory stimulation group mean  = .697, SEM  = .026; high sensory stimulation group mean  = .608, SEM  = .028), which became significant (*p*<.05), and the correlations between percentage retention at 7-day free recall and (i) proportion of hits ‘remembered’ (*p* = .1) and (ii) proportion of hits known (*p* = .1), which no longer reached significance. See File S1, including Table S1 in File S1, for the results of all repeated analyses.

#### Comments

The results of Experiment 1 demonstrate that the memory enhancement via wakeful rest can be observed via recognition, thus (i) confirming the feasibility of this paradigm for Experiment 2, and (ii) opening novel avenues for the examination of the memory boost and its cognitive basis. The latter will be discussed further in the Discussion.

## Experiment 2

The purpose of Experiment 2 was to examine whether a brief period of wakeful rest after new learning improves recognition memory, even for stimuli that cannot be rehearsed intentionally during the wakeful rest period. In everyday life people often learn new words that cannot be retrieved intentionally after a single exposure. For example, if one encountered someone with a foreign or unfamiliar name for the first time, one may not be able to recall their name freely. However, if the person's name was mentioned, one might recognize it as belonging to someone one has met before. Our paradigm was based on this scenario.

### Methods

The procedure of Experiment 2 was aligned as closely as possible to that of Experiment 1, but used recognition testing instead of free recall at both the 15-min and 7-day time points. In contrast to Experiment 1, there was no immediate test in Experiment 2: a test of immediate recognition would have introduced new stimuli, i.e. the foils, as well as taking up several minutes, and this could have interfered with the early consolidation of the word list, thus reducing the effect of minimal sensory stimulation shown in tests of free recall. Moreover, in contrast to Experiment 1, the stimuli used in Experiment 2 were non-words.

#### Participants

As in Experiment 1, we tested healthy elderly people. Fifty-four healthy elderly adults (21m/33f) were randomly assigned to one of two stimulation condition groups, the *high sensory stimulation* group (N = 27, mean age  = 72.22 years, age range = 61–89 years; NART-predicted IQ = 121.68, range = 112.98–127.02; 10m/17f) and the *minimal sensory stimulation* group (N = 27, mean age = 75.33 years, age range = 60–90 years; mean NART-predicted IQ = 120.9, range = 114.64–124.68; 11m/16f). As in Experiment 1, participants had no premorbid neurological or psychiatric history, and demonstrated normal scores on a thorough neuropsychological test battery, including the ACE-R [24], in which all participants scored ≥88 (high cut off). The groups did not differ significantly in age (*p* = .109), NART-predicted IQ (*p* = .268) or ACE-R scores (*p* = .349).

#### Design

Like Experiment 1, Experiment 2 included two testing sessions, Session 1 and Session 2, which were separated by 7 days. We used a mixed design to examine retention of a list of non-words. There were two key manipulations: stimulation condition (high sensory stimulation vs. minimal sensory stimulation; between subjects) and retention interval (15 minutes vs. 7 days; within subjects).

#### Materials

A total of 60 words were employed in this experiment, grouped into four lists of 15 words each. The four lists included the two lists used in Experiment 1 as well as two further lists. As in Experiment 1, the words were selected from the MRC Psycholinguistic database and were matched for number of letters, syllables, familiarity, concreteness, imaginability and frequency (frequency was taken from the British National Corpus). In order to obtain 60 non-recallable stimuli, each word was scrambled (e.g. Experiment 1 word ‘junction’  =  Experiment 2 word ‘toijcunn’). 30 of the scrambled non-words were used as targets, and the remaining 30 scrambled non-words were used as foils. Scrambled non-words had the same number of syllables as their English word counterparts to ensure consistency across experiments.

In order to avoid repetition of targets and foils in the 15-minute and 7-day tests, the target non-words were divided into two recognition tests, one for the 15-minute delay test, the other for the 7-day delay test: test A contained the target stimuli that had been presented in odd positions (word 1, word 3, word 5), and test B contained the target stimuli that had been presented in even positions (word 2, word 4, word 6). The order of tests A and B was counterbalanced across participants.

The 30 ‘spot-the-difference’ picture pairs from Experiment 1 were also used in Experiment 2 during filled delay periods (see Experiment 1 Methods for details).

#### Pilot investigations

Prior to Experiment 2, two pilot investigations were conducted in order to ascertain (i) that our non-words could not be retrieved intentionally, but (ii) that they could be recognised well in a Yes/No recognition test.

In the first pilot investigation (N = 12), participants listened to each of the 60 non-words and were asked to state if the items were in any way semantically meaningful to them. A semantic connection was made by one participant to four of the non-words, and these four non-words were re-scrambled and re-piloted in order to ensure that no semantic connection was made to any of the non-words.

In the second pilot investigation (N = 12), participants were presented with the 30 target non-words aurally. Immediately after presentation, participants were asked to recall as many of the non-words as possible, in any order. None of the participants were able to freely recall any of the non-words accurately, thus indicating that our lists of words were indeed non-recallable. Immediately after the free recall phase, participants completed a recognition test with the 30 target non-words and 30 foils. Participants had a mean d′ score of 1.44, showing that they were able to recognize the target non-words immediately after presentation (mean hit rate  = 0.73, mean false alarm rate  = 0.21), and thus that recognition testing could be conducted over longer time intervals.

#### Procedure

**Figure 4:**
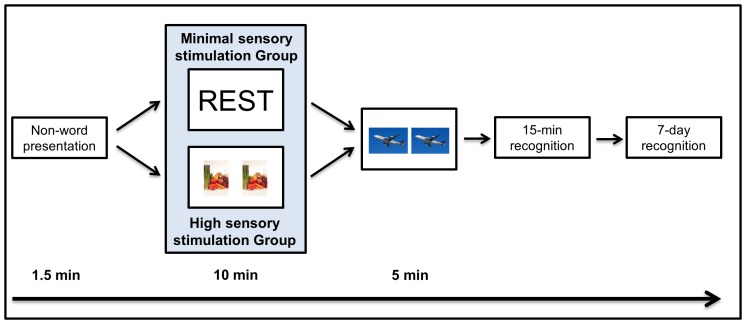
Participants were presented with 30 face/non-word name pairs at the beginning of Session 1, and instructed to try to remember as many non-word names as possible for subsequent recognition testing. Immediately after presentation, participants in the high sensory stimulation group completed a spot-the-difference task for 10 minutes, whereas participants in the minimal sensory stimulation group rested wakefully for 10 minutes. All participants then completed a 5-minute distractor task (spot-the-difference), which was succeeded by a 30-item (15 targets, 15 foils), yes/no non-word name recognition test, including a remember/know paradigm. 7 days later, participants completed a second and different 30-item (15 targets, 15 foils) yes/no non-word name recognition test, again including a remember/know paradigm.

Figure 4 illustrates the procedure of Experiment 2. In *Session 1* of Experiment 2, participants were presented with the 30 target non-words aurally. To provide context, the non-words were presented as peoples’ names, and were paired with a face taken from the Glasgow Unfamiliar Face Database (15 faces were female, 15 male). The face/name pairs were used to simulate a real life situation, i.e. meeting someone new with a foreign name. The following instructions were provided to all participants prior to the experiment: ‘I want you to imagine that you've moved to a new country and you've joined a bridge club. You're meeting the other club members for the first time, and they have names that sound foreign and unfamiliar to you. When you hear each name, you will see that person's face on the screen. I would like you to try to remember the club members’ names. After you've heard all of the names, you are going to be asked to identify them from a longer list of names, some of which you have heard before, and some of which you have not heard before. Your task will be to tell me whether or not you've met that person at the bridge club. You probably won't recognize all of the names, but do your best. Do you have any questions?’

The 30 face/name pairs were presented in the same random order across participants. As was the case for words in Experiment 1, non-words were presented aurally for a duration of one second each, with two seconds between non-words. Faces remained on the computer screen for the two second gap between each non-word (for a cumulative total of 3 seconds) to ensure equal timings across experiments.

After the 30 target face/name pairs were presented, participants in the minimal sensory stimulation group had a 10-minute delay of wakeful rest (see Experiment 1), while participants in the high sensory stimulation group completed 10 minutes of the spot-the-difference task (see Experiment 1). As in Experiment 1, both groups completed 5 minutes of the spot-the-difference task immediately after the post-learning delay to ensure that both groups engaged in the same activity prior to delayed recognition testing.

15 minutes after name/face presentation, participants completed a yes/no recognition test (*15-minute recognition test*). As in Experiment 1, target and foil words were presented aurally in the same random order for each participant. Participants heard the non-word names only (without face presentation), and were asked whether they had heard the name 15 minutes before. If they made an ‘old’ response, participants were asked to make a remember/know judgment (see Experiment 1) [25], [26]. Following the 15-minute recognition test, participants completed a post-experimental survey asking such questions as what they did during the wakeful rest delay (as applicable) and whether they thought about the non-words during the post-learning delay.

In *Session 2*, which occurred 7 days after Session 1, participants completed a second (and different) yes/no recognition test (*7-day recognition test*). Again, participants heard the non-word names, and were asked whether or not they heard the name 7 days before. If they made an ‘old’ response, a remember/know judgment was obtained as in Session 1.

Upon completion of the 7-day recognition test, participants were administered another post-experimental survey to ascertain whether they had thought about the non-word names or faces over the 7-day delay, and whether they expected to be asked about the names and/or faces again.

#### Scoring

As in Experiment 1, hit rates were calculated by dividing the number of targets correctly identified, by the total number of targets (/15). False alarm rates were calculated by dividing the number of foils incorrectly identified as targets, by the total number of foils (/15). These scores were used to calculate d-prime (d′) using the same formula as in Experiment 1. None of the participants had a hit rate of 1 or false alarm rate of 0, thus no corrections had to be made during the computation of d′. As in Experiment 1, we also calculated the correct response rate as a further measure of recognition accuracy ((hits + correct rejections)/30).

#### Statistical analyses

As in Experiment 1, the alpha level was set to.05 for all analyses, which were conducted in IBM SPSS Statistics 19. For our recognition accuracy measures (d′ and correct response rate) we carried out a mixed model, repeated measures ANOVA with within subjects factor time (15 minutes vs. 7 days) and between subjects factor group (high sensory stimulation vs. minimal sensory stimulation). Based on our previous findings [2], we ran two planned comparisons per recognition measure (d′, correct response rate, hit rate, false alarm rate, proportion of hits remembered, proportion of hits known), using one-way ANOVAs, to examine whether minimal sensory stimulation improved recognition performance (i) after 15 minutes and (ii) after 7 days. For the recognition accuracy measures we ran an additional planned comparison, based on previous findings [2], to examine whether 7-day recognition performance in the minimal sensory stimulation condition was equal or superior to 15-min recognition performance in the sensory stimulation condition. Lastly, we ran repeated measures ANOVAs to compare within each group the proportion of hits ‘remembered’ and the proportion of hits ‘known’ after 15 minutes and after 7 days.

### Results

Table 1 shows all data for the yes/no word recognition test (30 targets and 30 foils) after 15 minutes and after 7 days.

#### d′ prime

**Figure 5:**
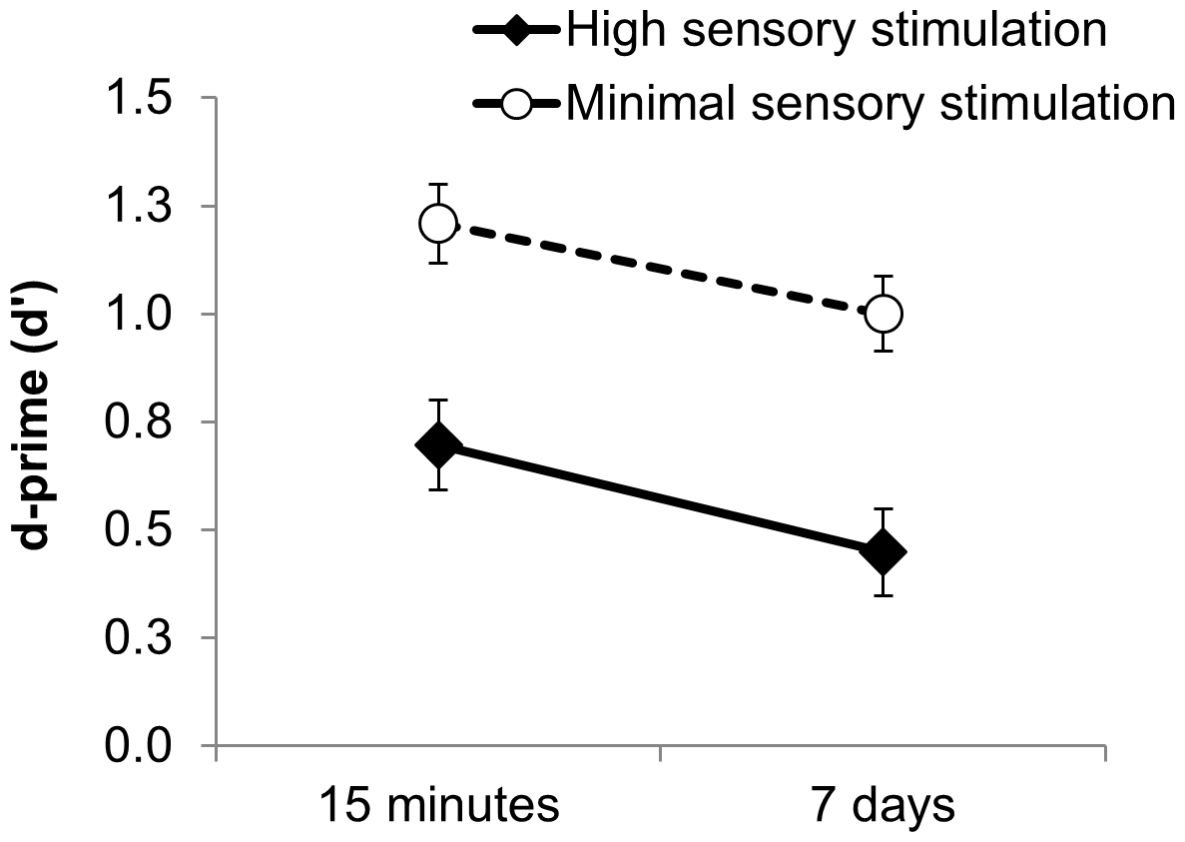
Mean d-prime (d)′ scores for the high sensory and minimal sensory stimulation groups after 15 minutes and after 7 days in Experiment 2. The minimal sensory stimulation group showed superior recognition performance relative to the high sensory stimulation group in a paradigm employing non-recallable stimuli after 15 minutes, and this benefit was maintained for 7 days. Error bars  =  standard error of the mean.

As shown in Figure 5, the 15-minute d′ score was significantly higher in the minimal sensory stimulation group than in the high sensory stimulation group, (*F*(1, 52) = 13.810, *p*<.001). In both groups the d′ score dropped significantly over the 7-day delay (*F*(1, 52) = 5.727, *p*<.05, η_p_^2^ = .099). However, the superior d′ score in the minimal sensory stimulation group relative to the high sensory stimulation group was maintained after 7 days (*F*(1, 52) = 17.345, *p*<.001) (see Figure 5 and Table 1), with no further additional benefit after 7 days, i.e. no significant group x time interaction (*F*(1, 52) = 0.04, *p* = .841, η_p_^2^ = .001, see Figure 5). As shown in Figure 5, *7-day* recognition (d′) of non-words learned prior to wakeful resting was higher than *15-minute* recognition (d′) of non-words learned prior to the spot-the-difference task (*F*(1, 52) = 5.080, *p*<.05).

#### Correct response rate

The main results for the correct response rates paralleled those of d′prime, as shown in Table 1 and by the absence of a significant group x time interaction (*F*(1, 52) = 0.08, *p* = .778, η_p_^2^ = .002). Moreover, as for d′, *7-day* correct response rate of non-words learned prior to wakeful resting was higher than *15-minute* correct response rate of non-words learned prior to the spot-the-difference task, and this difference was close to significance (*F*(1, 52) = 3.473, *p* = .068).

#### Hit rate and false alarm rate

As shown in Table 1, hit rate did not differ significantly between the high sensory stimulation group and the minimal sensory stimulation group after 15 minutes (*F*(1, 52) = 1.497, *p* = .227). After 7 days, a group difference emerged, although this did not reach significance (*F*(1, 52) = 2.910, *p* = .094). It should be noted however, that one participant in the high sensory stimulation group had a very high hit rate (0.9,>2.5 SD from group mean), coupled with a high false alarm rate (0.86), implying guessing. When this participant was removed from the analysis, the group difference in 7-day hit rate became significant (*F*(1, 51) = 4.741, *p*<.05). False alarm rate was significantly higher in the high sensory stimulation group than in the minimal sensory stimulation group after 15 minutes (*F*(1, 52) = 11.819, *p*<.005) and after 7 days (*F*(1, 52) = 10.581, *p*<.005). These results were unaffected when the aforementioned participant was removed from the analysis.

#### Remember/Know

There was no significant difference between the high sensory stimulation and minimal sensory stimulation group in the proportion of ‘remember’ responses for correctly identified targets, neither after 15 minutes (*F*(1, 52) = 1.324, *p* = .255) nor after 7 days (*F*(1, 53) = .362, *p* = .550). The same was true for ‘know’ responses. As shown in Table 1, in both groups there was a significantly lower proportion of ‘remember’ than ‘know’ responses after 15 minutes (minimal: *F*(1,26) = 15.526, *p*<.01, η_p_^2^ = .374; high: *F*(1, 26) = 43.275, *p*<.001, η_p_^2^ = .625) and after 7 days (minimal: *F*(1,26) = 24.806, *p*<.001, η_p_^2^ = .488; high: *F*(1, 26) = 45.635, *p*<.001, η_p_^2^ = .637).

#### Post-experimental questionnaire

The questionnaire data revealed the following: *Expected recall* - four participants in the high sensory stimulation group and 2 participants in the minimal sensory stimulation group reported that they had expected delayed recall in Session 2; *Delay activity and rehearsal* - no participants reported thinking about the words during the wakeful rest period. All participants reported mind-wandering. Moreover, no participants reported thinking about the words during the 7-day interval.

None of the results above changed when we repeated the analysis, including only those participants who did not report expecting delayed recall (high sensory stimulation group N = 23, minimal sensory stimulation group N = 25). See File S1, including Table S1 in File S1, for the results of all repeated analyses.

## Discussion

Our aim was to establish, via a controlled study, whether intentional rehearsal is *necessary* in order for a brief wakeful rest to boost recently acquired memories [1]–[3]. Our results suggest that this is not the case. Using non-recallable non-words to minimise potential intentional rehearsal, we show that a brief wakeful rest after learning improved recognition (d′ and correct response rate) after 15 minutes, and that this benefit was maintained over at least 7 days. In fact, as Figure 5 shows, *7-day* recognition (d′) of non-words learned prior to wakeful resting was higher than *15-minute* recognition (d′) of non-words learned prior to the spot-the-difference task.

Our manipulation was successful as evinced by our pilot study, demonstrating that none of our non-words could be recalled freely, even though the recall test occurred immediately after list presentation. This result was corroborated by the finding that none of the Experiment 2 participants thought about the non-word material during the wakeful rest period. Our findings thus refute the hypothesis that intentional rehearsal is necessary in order for a period of wakeful rest to boost recently acquired memory traces. Indeed, our findings suggest that, like sleep, wakeful rest alone can improve memory, without the contribution of intentional and/or conscious repetition or elaboration of recently acquired memories.

What is the cognitive basis of this memory boost via rest?

Given the design of our paradigm it is unlikely that this rest-related memory enhancement could be accounted for by reduced interference at *retrieval* following the rest delay, as compared to following the spot-the-difference delay. Retrieval interference, i.e. the competition between similar memory traces at retrieval, would have been minimal in both conditions given that the photos presented during the spot-the-difference task were of a different modality than the wordlists, and participants completed the task in silence [2], [3]. We acknowledge that some participants might have had an internal monologue while scanning the photos for differences, and that this could have produced a degree of verbal interference. However, the photos did not overlap semantically with any of the target words or foil words, certainly not with the non-words, all of which were deemed semantically meaningless by independent raters during piloting. Therefore, it is unlikely that any such internal monologue could have been sufficiently similar to the words/non-words to compete with subsequent word recall/recognition. Moreover, even if the spot-the-difference task had produced mild interference with retrieval, such interference should have been present in both groups, seeing as all participants completed a 5-minute distractor spot-the-difference task immediately before the 15-minute memory test (see Figures 1 and 4). However, the minimal sensory stimulation group outperformed the high sensory stimulation group in both free recall and recognition testing despite these common factors.

Our paradigm also rules out the possibility that wakeful rest had a mere ‘passive’ effect on new memory traces. This ‘passive’ hypothesis, originating from the sleep/memory field [7], [38], stipulates that wakeful rest passively protects new memories from new interfering information rather than actively promoting their consolidation [1], [7]. Therefore, according to this hypothesis, the benefit of wakeful rest is transient, lasting only until participants are exposed to interfering new information [1], [7]. Our findings are incompatible with this passive hypothesis. Specifically, the effect of wakeful rest was observed even though a 5-minute distractor task intervened between wakeful resting and 15-minute recall (see Figures 1 and 4), as observed also in related studies [2], [3]. More importantly, as found previously [2], the effect of wakeful rest was sustained over 7 days, which were filled with much activity and interfering new information. These findings of a lasting benefit, following further activity and information, cannot be accounted for by a passive, transient hypothesis of wakeful resting.

However, our finding of a *lasting*, benefit of wakeful rest, as compared to a *non-similar* delay task, can be accounted for straightforwardly by memory consolidation. Memory consolidation strengthens new memories over time [12] and is associated with the *spontaneous* reactivation of recent encoding-related neural activity [8]–[11], [15], [16]. Research suggests that this spontaneous reactivation occurs predominantly during states of relative immobility, such as sleep and *wakeful rest* [8], [9], perhaps due to the minimal amount of newly encoded information, which would otherwise hamper reactivation [2], [6], [7]. Therefore, it is hypothesised that periods of rest allow for more reactivations than do periods of sensory stimulation, thereby resulting in stronger memories [2], [7]. Indeed, recent human fMRI work shows that the degree of reactivation during rest is associated positively with subsequent memory [10], [11]. Our findings of a rest-related boost in free recall (Experiment 1) [2], [3] and recognition (Experiment 1 and 2) align closely with these recent developments in the memory consolidation literature.

It is of note that in contrast to free recall percentage retention, hit rate was not increased significantly by wakeful rest in our study (although after 7 days the group difference approached significance, and was significant after removal of the high hit rate + high false alarm outlier in Experiment 2). This more subtle rest effect in hit rate is likely due to the reduced sensitivity of hit rate to variations in memory strength above the critical threshold for an ‘Old’ response. Indeed, the more-fine grained analysis of hits via remember/know responses in Experiment 1 revealed a higher proportion of ‘remembered’ hits in the minimal sensory stimulation group than in the high sensory stimulation group (see Table 1 and Figure 3). This finding, which parallels similar remember/know results after sleep [39], is in keeping with the view that wakeful rest allowed for stronger/richer memories to be formed (it is beyond the scope of this paper to arbitrate between single-process theory vs. dual-trace theory interpretations of this effect). We do not interpret the absence of a significant wakeful rest effect on remember/know responses in Experiment 2 since meaningless non-words could not be connected well to existing memory representations during encoding, thus reducing the likelihood of ‘remember’ responses. Indeed, several participants attempted to connect the non-words to an English word or name that they knew, but found it very difficult to do so.

False alarms were consistently lower in the minimal sensory stimulation group than in the high sensory stimulation group, at all delays and in both Experiments (see Table 1). This finding corroborates recent reports of reduced false alarms (i) in humans following sleep, using the Deese-Roediger-McDermott (DRM) task [40], and following a caffeine-filled consolidation delay-period [41], and (ii) in memory-impaired rodents, following reduced sensory stimulation [42], [43]. The rest-induced reduction in false alarms observed here can be accounted for by superior memory consolidation, by means of the increased strength/quality bestowed on the target memory traces during wakeful resting: this increased strength/quality of the target memory traces could have allowed people to distinguish better whether or not a presented foil differed from one or more previous target memories, in particular where foils and targets overlapped to some degree. This interpretation of the reduced false alarms via rest is supported by the significant inverse correlation between false alarm rate and free recall percentage retention in Experiment 1, and by the ‘recall-to-reject’ literature, suggesting that sound/strong target memories are necessary for the correct rejection of foils [44], [45].

It is of note that, as shown in previous work [2], [3], the minimal sensory stimulation group was not completely immune to loss of word list material over the first 15 minutes. It is possible that the actual degree of benefit via wakeful rest was diminished in our study by the 5-minute distractor task that followed the 10-minute rest delay. However, a similar loss of word list material has been observed in a recent study, in which the rest delay (10 minutes) was followed immediately by the delayed recall test, without any intervening distractor task [3]. These findings suggest that some forgetting occurs, even over periods of minimal sensory stimulation. Given recent findings that autobiographical thinking can also interfere with word list consolidation [3], it is possible that the small drop in word list material during rest can be accounted for by this form of consolidation interference.

The present study and the previous free recall study [2] focused on healthy elderly people (≥60 years). However, the effect of wakeful rest on memory is not restricted to elderly people, as evinced by the finding of a robust effect of wakeful rest on the retention of common nouns in young people [1], [3]. This notwithstanding, the present recognition paradigm should be repeated in young people, in order to establish whether age affects the degree to which wakeful rest benefits recognition of common nouns and un-recallable non-words.

Our results indicate that the enhancement in memory via wakeful rest is *not* dependent upon intentional rehearsal of learned material during the rest period. We thus conclude that consolidation is *sufficient* for this rest-induced memory improvement to emerge. We propose that wakeful resting boosts memory consolidation, resulting in stronger and/or more veridical representations of experienced events, which can be detected both via tests of free recall and recognition, at least in healthy elderly people.

## Supporting Information

File S1 **Supporting Information for this article.** This file includes a full report of all Experiment 1 and Experiment 2 analyses, including only those participants who did not report thinking about the words (during the wakeful rest period and/or between sessions) or expecting delayed recall. Group means and SEMs are provided in Table S1 at the end of this file.
(DOCX)
