# Supplementary material for: Boosting Long-Term Memory via Wakeful Rest: Intentional Rehearsal Is Not Necessary, Consolidation Is Sufficient
Source: PLoS One. 2014 Oct 15;9(10):e109542. doi: 10.1371/journal.pone.0109542 (PMC4198139; doi:10.1371/journal.pone.0109542)
Supplement: File S1 — Supporting Information for this article. This file includes a full report of all Experiment 1 and Experiment 2 analyses, including only those participants who did not report thinking about the words (during the wakeful rest period and/or between sessions) or expecting delayed recall. Group means and SEMs are provided in Table S1 at the end of this file. (DOCX) [file pone.0109542.s001.docx]

**Supporting Information S1**

This file includes a full report of all Experiment 1 and Experiment 2 analyses, including only those participants who did not report thinking about the words (during the wakeful rest period and/or between sessions) or expecting delayed recall (Experiment 1: high sensory stimulation group N = 24, minimal sensory stimulation group N = 20; Experiment 2: high sensory stimulation group N = 23, minimal sensory stimulation group N = 25). Group means and SEMs are provided in Table S1 at the end of this document.

**Experiment 1**

Analyses repeated including only those participants who did not report thinking about the words (during the wakeful rest period and/or between sessions) or expecting delayed recall (high sensory stimulation group N = 24, minimal sensory stimulation group N = 20).

Results

**Immediate free recall*.*** Immediate recall scores did not differ between the minimal sensory stimulation group (mean = 6.15, SEM = .244) and the high sensory stimulation group (mean = 5.92, SEM = .232), (*F*(1, 42) = 0.476, *p* =.494). This indicates that baseline performance was matched for the stimulation condition groups.

**Delayed free recall - Retention of word lists after (a) 15 minutes and (b) 7 days*.*** 15-minute wordlist retention was significantly higher in the minimal sensory stimulation group (mean = 69.94, SEM = .02) than in the high sensory stimulation group (mean = 52.41, SEM = .032), (*F*(1, 42) = 20.129, *p* < .001). Retention dropped over 7 days in both stimulation groups (*F*(1, 42) = 99.621, *p* <. 001, η_p_^2^ = .703). However, the superior retention in the minimal sensory stimulation group (mean = 48.2, SEM = .027) relative to the high sensory stimulation group (mean = 28.16, SEM = .035) was maintained at 7-day delayed recall, (*F*(1, 42) = 19.556, *p* < .01), with no further additional benefit after 7 days, i.e. no significant time x group interaction (*F*(1, 42) = 0.296, *p*=.589, η_p_^2^ = .007).

Table S1 shows all data for the yes/no *word recognition* test (15 targets and 15 foils) taking place *after 7 days*.

**d’**. d’ was significantly higher in the minimal sensory stimulation group than in the high sensory stimulation group, (*F*(1, 42) = 11.443, *p* < .005).

**Correct response rate.** Correct response rate (hits + correct rejections/30) was significantly higher in the minimal sensory stimulation group than in the high sensory stimulation group (*F*(1, 42) = 13.819, *p* <.005).

**Hit rate and false alarm rate.** Hit rate was significantly higher in the minimal sensory stimulation group than in the high sensory stimulation group (*F*(1, 42) = 5.238, *p* < .05). Moreover, false alarm rate was significantly higher in the high sensory stimulation group than in the minimal sensory stimulation group (*F*(1, 42) = 4.234, *p* < .05).

**Remember/Know**. Table S1 shows that for hits, the proportion of ‘remember’ responses was significantly higher in the minimal sensory stimulation group than in the high sensory stimulation group (*F*(1, 42) = 6.042, *p* < .05). Correspondingly, the proportion of ‘know’ responses was significantly lower in the minimal sensory stimulation than in the high sensory stimulation group. Indeed, while in the minimal sensory stimulation group there was no significant difference in the proportion of ‘remember’ and ‘know’ responses (*F*(1, 19) = .001, *p* = .981, η_p_^2^ = 0), in the high sensory stimulation group, the proportion of ‘know’ responses was significantly higher than the proportion of ‘remember’ responses (*F*(1, 23) = 11.946, *p* < .005, η_p_^2^ = .342).

**Associations between 7-day free recall and 7-day recognition performance (collapsed over both groups).** Percentage retention at 7-day free recall correlated significantly and *positively* with d’ (*r* = .346, *p* < .05) and correct response rate (*r* = .400, *p* < .01). Moreover, percentage retention at 7-day free recall correlated significantly and *negatively* with false alarm rate (*r* = -.334, *p* < .05). However, percentage retention at 7-day free recall did not correlate significantly with hit rate (*r* = .168, *p* = .276), proportion of hits ‘remembered’ (*r* = .252, *p* =.1), or proportion of hits known (*r* = -.252, *p* =.1).

**Experiment 2**

Analyses repeated including only those participants who did not report thinking about the words (during the wakeful rest period and/or between sessions) or expecting delayed recall (Experiment 1: high sensory stimulation group N = 23, minimal sensory stimulation group N = 25).

Results

Table S1 shows all data for the yes/no word recognition test (30 targets and 30 foils) after 15 minutes and after 7 days.

**d’ prime*.*** The 15-minute d’ score was significantly higher in the minimal sensory stimulation group than in the high sensory stimulation group, (*F*(1, 46) = 9.563, *p* < .005). In both groups the d’ score dropped significantly over the 7-day delay (*F*(1, 54) = 5.933, *p* < .05, η_p_^2^ = .114). However, the superior d’ score in the minimal sensory stimulation group relative to the high sensory stimulation group was maintained after 7 days (*F*(1, 46) = 17.744, *p* < .001) (see Table S1), with no further additional benefit after 7 days, i.e. no significant group x time interaction (*F*(1, 46) = 0.412, *p* = .524, η_p_^2^ = .009). *7-day* recognition (d’) of non-words learned prior to wakeful resting was higher than *15-minute* recognition (d’) of non-words learned prior to the spot-the-difference task, and this difference was close to significance (*F*(1, 46) = 3.644, *p* = .063).

**Correct response rate.** The main results for the correct response rates paralleled those of d’prime, as shown in Table S1 and by the absence of a significant group x time interaction (*F*(1, 46) = 0.664, *p* = .419, η_p_^2^ = .014). Moreover, as for d’, *7-day* correct response rate of non-words learned prior to wakeful resting was higher than *15-minute* correct response rate of non-words learned prior to the spot-the-difference task, but this difference did not reach significance (*F*(1, 46) = 2.265, *p* = .139).

**Hit rate and false alarm rate.** As shown in Table S1, hit rate did not differ significantly between the high sensory stimulation group and the minimal sensory stimulation group after 15 minutes (*F*(1, 46) = .389, *p* = .536). After 7 days, a group difference emerged, although this did not reach significance (*F*(1, 46) = 3.061, *p* = .087). False alarm rate was significantly higher in the high sensory stimulation group than in the minimal sensory stimulation group after 15 minutes (*F*(1, 46) = 10.835, *p* < .005) and after 7 days (*F*(1, 46) = 10.146, *p* < .005).

**Remember/Know.** There was no significant difference between the high sensory stimulation and minimal sensory stimulation group in the proportion of ‘remember’ responses for correctly identified targets, neither after 15 minutes (*F*(1, 46) = 1.854, *p* = .180) nor after 7 days (*F*(1, 46) = 1.113, *p* = .297). The same was true for ‘know’ responses. As shown in Table S1, in both groups there was a significantly lower proportion of ‘remember’ than ‘know’ responses after 15 minutes (minimal: *F*(1,24) = 12.111, *p* < .005, η_p_^2^ = .335; high: *F*(1, 22) = 36.418, *p* < .001, η_p_^2^ = .623) and after 7 days (minimal: *F*(1,24) = 19.625, *p* < .001, η_p_^2^ = .450; high: *F*(1, 22) = 42.016, *p* < .001, η_p_^2^ = .656).

**Table S1.**

**Mean recognition performance (+ SEM) of the minimal sensory stimulation groups (wakeful rest delay) and the high sensory stimulation groups (spot-the-difference delay) in Experiment 1 and 2, including only those participants who did not report thinking about the words (during the wakeful rest period and/or between sessions) or expecting delayed recall (Experiment 1: high sensory stimulation group N = 24, minimal sensory stimulation group N = 20; Experiment 2: high sensory stimulation group N = 23, minimal sensory stimulation group N = 25).**

| **Experiment 2** | | | | | | | |  | **Experiment 1** | | |
| --- | --- | --- | --- | --- | --- | --- | --- | --- | --- | --- | --- |
| (non-words) | | | | | | | |  | (common nouns) | | |
|  | **15 minutes** | | |  | **7 days** | | |  | **7 days** | | |
|  | **Minimal** | **High** | ***p-*value** |  | **Minimal** | **High** | ***p-*value** |  | **Minimal** | **High** | ***p-value*** |
| **Yes/No Test** |  |  |  |  |  |  |  |  |  |  |  |
| **d'** | **1.202 (.096)** | **.739 (.116)** | **< .005** |  | **1.016 (.089)** | **.420 (.112)** | **< .001** |  | **1.078 (.089)** | **.636 (.093)** | **< .005** |
| **Correct response rate** | **.711 (.015)** | **.633 (.020)** | **< .005** |  | **.672 (.016)** | **.568 (.017)** | **< .001** |  | **.698 (.016)** | **.614 (.016)** | **< .005** |
| **Hit rate** | .672 (.027) | .643 (.037) | .536 |  | .549 (.038) | .455 (.038) | .087 |  | **.697 (.026)** | **.608 (.028)** | **< .05** |
| **False alarm rate** | **.252 (.023)** | **.377 (.031)** | **< .005** |  | **.205 (.020)** | **.320 (.031)** | **< .005** |  | **.300 (.022)** | **.381 (.030)** | **< .05** |
|  |  |  |  |  |  |  |  |  |  |  |  |
| **Remember/Know** |  |  |  |  |  |  |  |  |  |  |  |
| **Proportion Remember - Hits** | .359 (.041) | .285 (.036) | .180 |  | .316 (.042) | .256 (.038) | .297 |  | **.499 (.040)** | **.355 (.042)** | **< .05** |
| **Proportion Know - Hits** | .641 (.041) | .715 (.036) | .180 |  | .684 (.042) | .744 (.038) | .297 |  | **.501 (.040)** | **.646 (.042)** | **< .05** |
|  |  |  |  |  |  |  |  |  |  |  |  |
| **Proportion Remember - False alarms** | .086 (.028) | .065 (.028) | .603 |  | .141 (.050) | .090 (.049) | .473 |  | .085 (.020) | .023 (.014) | .875 |
| **Proportion Know - False alarms** | .914 (.028) | .936 (.028) | .603 |  | .860 (.050) | .910 (.049) | .473 |  | .973 (.085) | .977 (.067) | .875 |
